# Testing entanglement of annihilation photons

**DOI:** 10.1038/s41598-023-34767-8

**Published:** 2023-05-09

**Authors:** Alexander Ivashkin, Dzhonrid Abdurashitov, Alexander Baranov, Fedor Guber, Sergey Morozov, Sultan Musin, Alexander Strizhak, Igor Tkachev

**Affiliations:** 1 Institute for Nuclear Research RAS, Moscow, 117312 Russia; 2 National Research Nuclear University MEPhI, Moscow, 115409 Russia; 3 Moscow Institute of Physics and Technology, Moscow, 141701 Russia

**Keywords:** Quantum mechanics, Single photons and quantum effects

## Abstract

We present a new experimental study of the quantum entanglement of photon pairs produced in positron-electron annihilation at rest. Each annihilation photon has an energy that is five orders of magnitude higher than the energy of photons in optical experiments. It provides a unique opportunity for controlled Compton pre-scattering of initial photons before the polarization measurements. The experimental setup includes a system of Compton polarimeters to measure the angular correlations of annihilation photons in initial and thus prepared pre-scattered states. For the first time, a direct comparison of the polarization correlations of initial and pre-scattered annihilation photons has been carried out. The angular distributions of scattered in polarimeters photons turned out to be the same for both types of events. Moreover, the correlation function in the Bell’s inequality is also the same for both states. We discuss the implications of our results for quantum measurement theory and for the quantum-entangled positron emission tomography.

## Introduction

The entanglement of a quantum system is a simple consequence of the superposition principle and means that the state of the system cannot be represented as a product of the states of individual subsystems. Historically, however, this term was introduced by Schrödinger, referring to the entanglement of our knowledge of quantum systems^1,2^. Such an interpretation of entanglement is quite relevant for the current situation with a system of two photons formed by positron-electron annihilation at rest. The description of this system has been the subject of pioneering work on quantum entanglement in the past century and, as we will see below, is still unclear in some respects.

The study of this two-photon system has a long and remarkable history, which can be divided into several stages. Initially, the idea of measuring a pair of annihilation photons was proposed in 1946 by Wheeler^3^. He considered at that time a hypothetical bound system of an electron and a positron with an orbital momentum equal to one or zero. In the latter case, due to the conservation of angular momentum and parity, the photons of the pair formed during positron-electron annihilation have mutually perpendicular polarization. In the same article, in order to test the predicted correlation of photon polarization, Wheeler proposed an experimental scheme with two Compton scatterers and detectors of scattered photons, which has already become a classic. Since photons are scattered predominantly perpendicular to the polarization plane, the dependence of the number of registered photons on the angle between the scattering planes should have a maximum and minimum at $90^\circ$ and $0^\circ$, respectively.

Almost simultaneously, two theoretical papers by Pryce and Ward^4^ and by Snyder et al.^5^ predicted the behavior of the angular distribution of scattered annihilation gamma rays. It was obtained on the basis of the Klein-Nishina formula^6^ using the maximally entangled pure state as the initial one:

1 $$\begin{aligned} {\Psi } = \frac{1}{\sqrt{2}}( {|{{H_1}{V_2}}\rangle } + {|{{V_1}{H_2}}\rangle }), \end{aligned}$$

where H(V) represent the horizontal (vertical) linear polarization of the first or second photon. In this state, photons do not have a definite polarization, albeit their polarizations are mutually orthogonal. It was shown that the ratio $R = {N_{\perp }}/{N_{\parallel }}$ of the number of counts for perpendicular and parallel orientations of scattering planes reaches a maximum of $R=2.85$ in the case when photons are scattered at an angle of $82$° to their initial momenta. These predictions were brilliantly confirmed in a pioneering experiment by Wu and Shaknov^7^. Their experimental ratio $R=2.04\pm 0.08$ was consistent with the theory, taking into account the finite solid angles of the detectors.

Seven years later, the results obtained were seriously rethought in the paper by Bohm and Aharonov^8^, devoted to the experimental verification of the famous EPR paradox by Einstein, Podolsky and Rosen^9^. The authors emphasized that positron-electron annihilation with zero orbital angular momentum produces two photons described by the *entangled* wave function, Eq. (1), and therefore provides a particular entangled system for studying the EPR paradox. The authors calculated ratio *R* and the angular distributions of Compton scattered photons for several types of initial quantum states. They confirmed $R\approx 2.85$ for the state Eq. (1) and concluded that $R=1$ for a separable mixed state of annihilation photons described by the density matrix:

2 $$\begin{aligned} \rho = \frac{1}{2}( {|{{H_1}{V_2}}\rangle }{\langle {{H_1}{V_2}}|} + {|{{V_1}{H_2}}\rangle }{\langle {{V_1}{H_2}}|}). \end{aligned}$$

According to the author’s statement, the measured ratio^7^ $R=2.0$ is experimental evidence of distant correlations leading to the EPR-paradox. Bohm and Hiley^10^ argued that measuring one of the two initial photons leads to the collapse of the entangled state Eq. (1) to the separable state described by Eq. (2).

It is worth mentioning that the experimental verification of entanglement was first discussed seven years before the appearance of famous Bell’s theorem^11,12^, which is basic instrument of modern experimental tests of entanglement.

In subsequent years a series of experiments^13–18^ was performed to measure the ratio *R* with better accuracy. The most precise results were obtained by Langhoff^13^ ($R=2.47\pm 0.07$) and by Kasday et al.^15^ ($R=2.33\pm 0.10$). The geometrical corrections that account for the finite solid angles of detectors provided the results consistent with the theoretically predicted value $R=2.85$.

Quite recently Caradonna et al.^19^ derived the cross-sections for Compton scattering of annihilation photons in several (maximally entangled and separable mixed) states using the matrix representation of the Klein-Nishina formula. They confirmed the theoretical results^8,10^ and concluded that the previous experimental measurements prove the entanglement of system of two initial annihilation photons.

Strong polarization correlations for annihilation photons in maximally entangled states and the theoretically predicted absence of correlations for separable states motivated the development of a new generation of Positron Emission Tomography (PET), the so-called quantum-entangled QE-PET with Compton scattering reconstruction^20,21^. Attention is paid to the kinematic restrictions on the angular distributions of scattered gammas in attempts to suppress the scatter and random backgrounds and improve the quality of PET images. In recent years, significant efforts have been made to create working prototypes of PET using the reconstruction of the Compton scattering kinematics. More recently, the research Jagiellonian Positron Emission Tomograph (J-PET)^22,23^ was built of plastic scintillators. Identification of double Compton scattering of photons in J-PET plastic bars makes it possible to determine the linear polarization of primary photons and to measure the angular correlations.

The verification of entanglement mentioned above relies exclusively on the angular correlations of scattered annihilation photons. One might wonder why Bell’s theorem^11,12^, or the Clauser-Horne-Shimony-Holt (CHSH) inequality^24^ as a particular practical case, has not been applied here. The explanation is quite simple and is related to the low efficiency of Compton polarimeters, which are the only tool for measuring the polarization of high-energy gamma rays. This efficiency is strongly dependent on the energy of gamma-ray and is always less than 0.7 for annihilation photons with an energy of 511 keV. This issue has been discussed by Clauser and Shimony^25^, who pointed out that entanglement in positron-electron annihilation can only be demonstrated if the polarimeter efficiency is greater than 0.83. In this case, the *S*-function in the CHSH inequality, reduced to the product of the efficiencies of two Compton polarimeters, would exceed the required limit of 2. In reality, for annihilation photons, this S-function can only reach a maximum of 1.4, since the product of two efficiencies is less than 0.5.

Nevertheless, the *S*-function was measured in 1996 by Osuch et al.^26^. They counted the number of scattered photons in Compton polarimeters located at different azimuthal angles. The correlation coefficients were obtained from the coincidence of the count rates between two sets of polarimeters installed on opposite sides of the annihilation photon source. The *S*-function constructed from these coefficients perfectly reproduces the theoretically predicted behavior:

3 $$\begin{aligned} S = - p_0 (3\cos (2\phi ) - \cos (6\phi )), \end{aligned}$$

where $\phi$ is the azimuthal angle between the polarimeters, and $p_0$ is the product of the efficiencies of two opposite Compton polarimeters. Of course, the resulting maximum value of *S* was well below the required limit of 2. Nevertheless, subsequent corrections for the measured polarimeters efficiencies allowed the authors to state that the CHSH inequality is violated by twelve standard deviations.

The agreement of numerous experimental results with the predictions of quantum theory gives the impression of a complete understanding of the behaviour of a system of two annihilation photons. However, the situation became rather uncertain in 2019 with the appearance of the theoretical work of Hiesmayr and Moskal^27^, who applied an open quantum formalism to the Klein-Nishina formula and obtained the same Compton scattering cross section for both maximally entangled and separable states of annihilation photons. Since the cross sections of any reactions are of fundamental meaning, their identity for considered quantum states leads to the same angular correlations of scattered photons. This result fundamentally contradict previous theoretical considerations^8,10,19^ and, therefore, claims the incompleteness of existing experimental studies, which are based on the assumed difference in the Compton scattering of photon pairs in maximally entangled and mixed states.

The results in ref.^27^ obviously affect the possibility of using quantum entanglement in the development of PET imaging, since it is based on the expectation that parasitic Compton scattering of the initial photon in the body causes the transition of the initial maximally entangled state to a separable one. To resolve apparent theoretical contradictions, Watts et al.^28^ built a prototype PET with modern semiconductor gamma detectors and a passive Compton scatterer for one of the annihilation photons. They measured the angular correlation of scattered photons for two types of events: initial photons without a passive scatterer and pre-scattered events with a passive scatterer placed in the path of one of the annihilation photons. According to authors, the decohering process in the passive scatterer leads to a separable state with lost entanglement. Unfortunately, the low sensitivity to the measured polarization and large statistical measurement errors did not allow to draw an unambiguous conclusion. New dedicated measurements with intact and pre-scattered states of annihilation photons are required to solve the theoretical puzzle. For this purpose, we have developed a setup^29^ of Compton polarimeters with a large solid angle and an active intermediate Compton scatterer, which has been operating for about a year and a half at INR RAS. In this paper, we report our measurements of polarization correlations in Compton scattering of initial and pre-scattered pairs of annihilation photons.

## Methods

**Figure 1:**
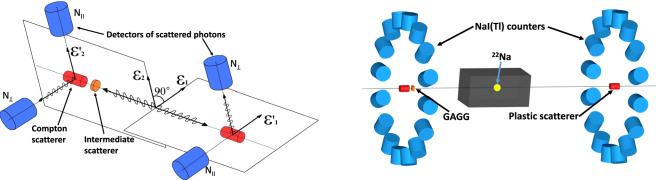
Left—principal scheme for measuring polarization correlations of initial and prepared pre-scattered states of annihilation photons. It includes two Compton polarimeters and an intermediate scatterer. Each Compton polarimeter consists of a scatterer and two orthogonal detectors of scattered gammas. $\varepsilon _1$ ($\varepsilon _2$) and $\varepsilon _1'$ ($\varepsilon _2'$) are the polarization vectors of the initial and scattered gammas, respectively. $N_{\parallel }$ and $N_{\perp }$ denote detectors parallel (perpendicular) to the initial polarization vector. Right—scheme of a two-arm experimental setup. Each arm consists of a plastic scatterer on the setup axis and 16 NaI(Tl) counters orthogonal to the axis. The $^{22}$Na source of positrons is placed in a lead collimator between the arms closer to intermediate GAGG scatterer for preparing pre-scattered annihilation photons.

The principles for measuring the polarization correlations of annihilation photons are illustrated in Fig. 1, left. Two Compton polarimeters are required for measuring both photons with opposite momenta. Each polarimeter consists of a Compton scatterer and two detectors of scattered gammas arranged orthogonally. An intermediate scatterer is placed in the path of one of the initial photons to create a tagged subset of pre-scattered events.

Each annihilation photon has an energy equal to the electron mass (511 keV), which is five orders of magnitude higher than the energy of optical photons. Such a sharp difference has both advantages and disadvantages in measuring polarization states. The need to use Compton polarimeters with a relatively low efficiency has already been discussed and makes it problematic to use the CHSH inequality for direct testing the photons entanglement. At the same time, due to the high energy of annihilation photons, one can arrange their interaction/measurement with environment without strong degradation of initial two-photon kinematics. Really, the Compton scattering at any, even a sufficiently small angle, leads to the energy loss of the initial photon. Thus, annihilation photons provide a unique opportunity for direct comparison of polarization correlations for both initial and pre-scattered events, where some energy fraction of initial photon is transferred to the recoil electron.

The measurements are based on the dependence of the differential cross section of Compton scattering on the polarization direction, which is given by the well-known Klein-Nishina formula^6^:

4 $$\begin{aligned} \frac{d\sigma }{d\Omega }=\frac{ (r_e\varepsilon )^2}{2}\cdot \left( \varepsilon +\frac{1}{\varepsilon }-2\sin ^2{\theta }\cdot \cos ^2{\phi } \right) , \end{aligned}$$

where ${r_e}$ is the classical electron radius, $\varepsilon \equiv E_1/E$, *E* ($E_1$) is the energy of the incident (scattered) photon, $\theta$ is the scattering angle and $\phi$ is the angle between the scattering plane and the direction of polarization of the incident photon. The scattered photon energy is ${E_1=E\cdot m_e/(m_e+E\cdot (1-\cos {\theta })})$, where $m_e$ is the electron mass. As follows from Eq. (4), photons scatter predominantly orthogonally to the polarization plane.

Like optical polarimeters, the Compton polarimeter has two main components. Namely, the Compton scatterer measures the polarization of photons in place of a conventional polaroid, and the detectors of scattered photons play the role of photodetectors.

The main characteristic of a Compton polarimeter, which determines the sensitivity to the measured polarization, is the analyzing power (efficiency of polarimeter): $A(\theta )=\frac{N_\perp -N_\parallel }{N_\perp +N_\parallel }$, where ${N_\perp }$ $({N_\parallel })$ denotes the number of registered events in counters located perpendicular (parallel) to the polarization of the incident photons. Equivalently, at large statistics,

5 $$\begin{aligned} A(\theta )=\frac{\frac{d\sigma }{d\Omega }(\theta ,\phi =90^\circ )-\frac{d\sigma }{d\Omega }(\theta ,\phi =0^\circ )}{\frac{d\sigma }{d\Omega }(\theta ,\phi =90^\circ )+\frac{d\sigma }{d\Omega }(\theta ,\phi =0^\circ )}. \end{aligned}$$

Using Eq. (4), the analyzing power is obtained as^30^:

6 $$\begin{aligned} A(\theta )=\frac{\sin ^2{\theta }}{E_{1}/E+E/E_{1}-\sin ^2{\theta }}. \end{aligned}$$

As follows from Eq. (6), for a given energy of the initial gamma, the analyzing power depends on the scattering angle. For completely polarized photons with an energy of 511 keV, the analyzing power reaches a maximum of $A=0.69$ at $\theta =82^\circ$ and equals $A=0.67$ for right scattering angle.

In the case of an entangled pair of photons with mutually orthogonal polarizations, the probability of Compton scattering at scattering angles $\theta _1, \theta _2$ is given by the following expression^5,19,27^:

7 $$\begin{aligned} P(\Delta \phi )={k_1 k_2 \left( 1-\alpha (\theta _1)\alpha (\theta _2)\cos (2\Delta \phi )\right) }, \end{aligned}$$

where $k_1$, $k_2$ are the kinematic factors for the first and second scattered photons, $\Delta \phi$ is the angle between scattering planes, the parameters $\alpha (\theta _1)$ and $\alpha (\theta _2)$ are given by Eq. (6) and coincide with the analyzing power of the corresponding Compton polarimeter. The product of the analyzing powers is equal to the modulation factor^31^ $\mu$ which determines the sensitivity of a setup to the measured polarization. Therefore, Eq. (7), together with the dependence of photon counts on the azimuthal angle, also gives the modulation factor $\mu$ of the experimental setup.

Based on these principles an experimental setup^29^ was constructed to measure the polarization correlation of annihilation photons in both initial and decoherent states. The setup comprises two equivalent arms of Compton polarimeters and a $^{22}$Na positron source placed between these arms, as shown in Fig. 1, right. A source of positrons with an activity of $\sim 50$ MBq was fabricated by irradiating a 1 mm thick aluminum plate with 130 MeV protons at the INR RAS isotope facility^32^. The source is located in a horizontal hole with diameter of 5 mm in a lead cube providing collimated beams of annihilation photons in opposite directions from the source. The positrons from $^{22}$Na source almost immediately thermalize in aluminium and annihilate with electrons with zero relative angular momentum^33^. According to the conservation laws of angular momentum and parity the obtained annihilation photons must be in maximally entangled state^5,8,10^.

Each arm of the setup consists of a plastic scintillation scatterer and a ring of 16 NaI(Tl) scintillation detectors of scattered photons with an azimuthal angle between adjacent detectors of $22.5^{\circ }$. NaI(Tl) counters detect photons scattered at an angle of about $90^{\circ }$. Each pair of orthogonal NaI(Tl) counters and a plastic scatterer of the same arm form an elementary Compton polarimeter. Due to the azimuthal symmetry of the setup, each NaI(Tl) counter registers the vertical or horizontal polarization component depending on the orientation of the scattering plane. The chosen layout leads to a compensation for possible systematic errors caused by different efficiencies and inaccuracies in the positions of the NaI(Tl) counters.

The distance between the plastic scatterers is about 70 cm. To produce the pre-scattered photons in a subset of events, an intermediate scatterer , a gadolinium-aluminum-gallium garnet (GAGG) scintillator, with the thickness of 7 mm and transverse dimensions of 15 × 15 mm$^2$ is located at a distance of 1 cm from one of the plastic scatterers. The $^{22}$Na positron source is located 10 cm closer to the arm with the GAGG scintillator to ensure that the first photon interaction occurs in the intermediate scatterer. More detailed information about the setup geometry is available in ref.^29^.

**Figure 2:**
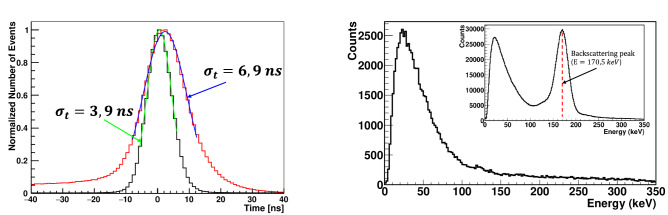
Left—time coincidence spectra between signals in intermediate GAGG and plastic scatterers. Red and black lines correspond to events with energy release in GAGG in the ranges of 2–40 keV and 40–120 keV, respectively. Blue and green lines are the results of the Gaussian fit of the corresponding distributions. The numbers indicate the time resolution for these two cases. Right—the energy spectra in GAGG scatterer for events within the true time coincidence peak. Here, events are selected that hit the NaI(Tl) counters. Insert shows the extended GAGG energy spectrum for all events, regardless of the hits in NaI(Tl) counters. The prominent peak at 170.5 keV corresponds to photons backscattered by the adjacent plastic scatterer and absorbed by GAGG. This peak is used for energy calibration of the intermediate scatterer.

The GAGG scatterer is the key element that separates events into two subsets with initial or pre-scattered photons. Therefore, the reliability of interaction identification in the intermediate scatterer is the most important feature of the setup. The timing and amplitude of the signals are used to identify the interaction in the GAGG scintillator and are shown in Fig. 2.

The time coincidence spectra of signals from intermediate and plastic scatterers are presented for two cases, with low and high energy deposition in the GAGG. In the first case, the scattering angles in GAGG are small and the influence on the momentum of the initial annihilation photon is minimal. In these events, the noise of the photodetector and readout electronics more strongly affects the time resolution compared to events with higher energy deposition.

Figure 2, on the right, shows the energy spectra in the intermediate scatterer for events in true time coincidence window with hits in the NaI(Tl) counters. The high light yield of GAGG^34,35^ makes it possible to detect recoil electrons with an energy threshold of 2 keV. The permanent energy calibration of GAGG is performed by detecting 170.5 keV energy deposition from photons backscattered by an adjacent plastic scatterer and absorbed in GAGG.

The event is recorded in case of coincidence of signals in two plastic scatterers. Such loose event selection allows permanent calibration of detectors and study of background conditions. The photon detection in NaI(Tl) counters and/or in GAGG scatter is identified during the off-line analysis after time and amplitude calibration of all active elements in setup. We define the tagging of events of various types analyzed in polarimeters as follows. A pair of photons is considered in initial state if no interaction is observed in the intermediate GAGG scatterer. Otherwise, the detected energy in the GAGG scatterer means that the photons have undergone pre-scattering.

The sensitivity of the setup to polarization measurements was studied by the Monte Carlo simulation using the latest version of Geant4^36,37^ particle simulation framework, where the theoretical formula Eq. (7) is implemented for initial annihilation photons. Since the NaI(Tl) detectors are located orthogonally to the Compton scatterers, the theoretical ratio of counts for perpendicular and parallel orientations of the scattering planes can be $R=2.6$ which is less than the maximum $R=2.85$ for the optimal $82^\circ$ scattering angle. In a setup with a realistic detector geometry, photons are detected in the range of scattering angles of $80^\circ{-}100^\circ$, which leads to an additional reduction to $R=2.40$. The modulation factor for entangled photons is equal to $\mu =0.41$, which is about 9% less than the theoretical value $\mu =0.44$ for the right scattering angle.

Monte Carlo simulations estimated also the contribution of systematic errors caused by possible counters inefficiencies and inaccuracies in the positions of the detectors and the positron source. Due to the azimuthal location of the detectors and cancellation of geometrical effects, systematic errors turned out to be almost an order of magnitude smaller than the statistical errors achieved and were not taken into account. For measurement results below, only statistical errors are shown.

## Results

### Experimental spectra

**Figure 3:**
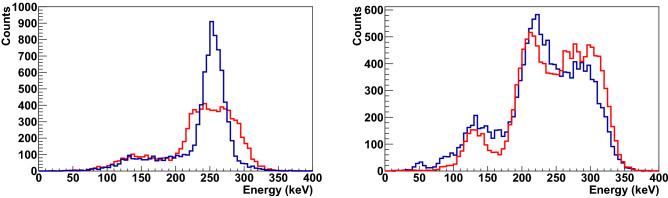
Left—energy spectra in NaI(Tl) counters for events in initial states without energy deposition in the intermediate GAGG scatterer (blue line) and for pre-scattered events with energy deposition in the intermediate GAGG scatterer below 30 keV (red line). Right—the energy spectra in NaI(Tl) counters for pre-scattered photons with energy deposition in the intermediate GAGG scatterer between 30 and 110 keV. The blue line is the experimental data. The red line is the result of a Monte Carlo simulation using the ideal energy resolution of the NaI(Tl) counters.

Identification of the Compton scattering kinematics is carried out by monitoring the energy release in all active elements of Compton polarimeters, namely, in NaI(Tl) detectors of scattered photons, plastic scatterers, and the intermediate GAGG scatterer. Scattering of the initial annihilation photons at $90^{\circ }$ releases an equivalent energy of 255 keV both in the plastic scatterer and in the NaI(Tl) counter. According to Monte Carlo simulations, in a setup with a realistic detector geometry, photons are detected in the range of scattering angles of $80^\circ{-}100^\circ$ with energy deposition in NaI(Tl) from 235 to 280 keV. This is illustrated in Fig. 3, which shows energy deposition spectra in NaI(Tl) detectors for various Compton scattering kinematics.

The relatively narrow NaI(Tl) energy peak for initial states reflects the range of scattering angles of detected photons. The situation is completely different for pre-scattered events after the interaction of photons with an intermediate scatterer. Even a few percent energy loss (below 30 keV) of the original 511 keV photons in the GAGG scintillator leads to a significant distortion of the energy spectrum. This is the effect of double Compton scattering of the initial annihilation photon in both GAGG and plastic scintillators. After the first scattering in GAGG, the photon deviates within a few degrees from its original direction. Subsequent interaction in a plastic scintillator increases the ranges of scattering angles and energy deposition of photons registered in NaI(Tl).

**Figure 4:**
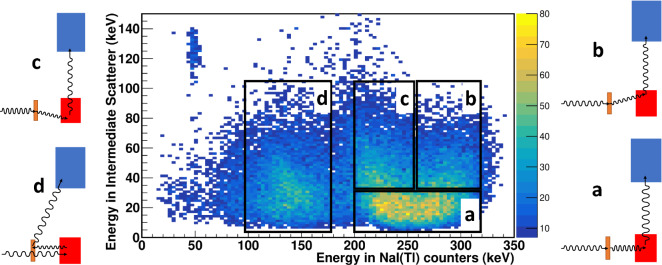
Correlation between energy depositions in the intermediate GAGG scatterer and in NaI(Tl) counters. Several groups of events in black boxes marked as “a”,“b”, “c” and “d” can be distinguished. They correspond to different Compton scattering kinematics in the GAGG scintillator, shown in the diagrams to the left/right of the correlation plot. The brown, red and blue boxes in the diagrams mark the intermediate GAGG scatterer, the plastic scatterer and the NaI(Tl) counter, respectively.

Events with a higher energy deposition in the intermediate scatterer (up to 110 keV) form a complex structure in the NaI(Tl) spectrum. Visible peaks in this energy spectrum correspond to particular cases of the kinematics of Compton scattering. The Monte Carlo simulation of the double Compton scattering of 511 keV photons confirmed that the observed structure in the NaI(Tl) spectrum reflects the kinematics of the first scattering in the GAGG scintillator, which leads to distinct groups of events. These particular cases of Compton scattering and the correlation between energy depositions in the NaI(Tl) and GAGG scintillators are shown in Fig.  4.

In this figure, several distinct groups of events can be identified, labeled as classes “a”, “b”,“c”, and “d”. The double Compton scattering kinematics for these events can be determined using a simple analytical calculation or Monte Carlo simulation. Group “a” represents scattering processes with an electron recoil energy in the GAGG scintillator below 30 keV and, accordingly, with the smallest scattering angles.

The range of scattering angles by the GAGG in the “b” and “c” regions is identical and it is larger as compared to the “a”-events. However, events in the “b” region have maximum energy deposition in NaI(Tl) detectors from two sequential Compton scatterings with the first scattering in the GAGG in the direction of the NaI(Tl) counter. In contrast to these events, the “c” group represents a double Compton scattering with the sum of scattering angles exceeding $90^\circ$, since the first scattering in the GAGG scintillator deflects the photon in the opposite direction from the NaI(Tl) counter. Therefore, more energy is left in the plastic scintillator and less for the NaI(Tl) detector remains.

The most complex kinematics of double Compton scattering is observed for the “d” group of events with the lowest energy registered by NaI(Tl). The original annihilation photons in these events pass through the GAGG scintillator without interaction and are backscattered in the plastic scatterer. The second scattering at about $90^\circ$ occurs in the GAGG scintillator, followed by photon registration in NaI(Tl) counters. Unlike other classes of events with relatively small scattering angles in GAGG, the “d” group represents Compton scattering at maximum angles about $180^\circ$ and with maximum energy losses in plastic scatterer.

### Angular distributions of scattered photons.

Previous experiments^13–18^ with annihilation photons have mainly measured the dependence of the number of counts of scattered photon detectors on the azimuthal angle between these detectors. This dependence is described by Eq. (7) and has a cosine-like behaviour. As follows from the formula, the ratio of counts *R* for perpendicular and parallel orientations of the scattering planes reaches a maximum of $R=2.85$ if both photons are scattered at an angle of 82° to their initial momenta. According to Eq. (7), the number of counts in detectors of scattered photons can be approximated as:

8 $$\begin{aligned} N(\phi )=A-B\cos (2\Delta \phi ), \end{aligned}$$

where $\Delta \phi$ is the angle between scattering planes, A and B are the fit constants. As follows from Eqs. (7) and (8), the ratio *R* equals $R=({A+B})/({A-B})$, while the modulation factor is $\mu =B/A$.

**Figure 5:**
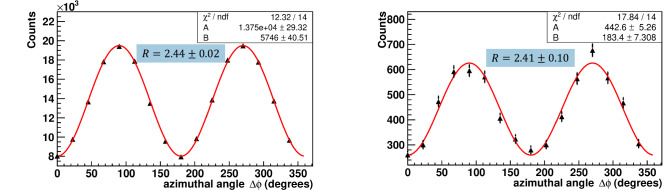
Dependence of coincidence counts in NaI(Tl) detectors on the azimuthal angle between these detectors for initial states (left) and events with pre-scattered in GAGG scintillator photons (right). The solid line corresponds to the fitting function Eq. (8). The numbers in the blue area on the graphs indicate the *R* ratio for the corresponding class of events.

In our experiment, the data set without energy deposition in intermediate GAGG scatterer is associated with initial photons, Eq. (1). In this case, the modulation factor coincides with the product of the analyzing powers of the corresponding Compton polarimeters, $\mu =\alpha ({\theta _1})\cdot \alpha ({\theta _2})$, see Eq. (7). Other events with interaction in the GAGG scatterer correspond to pre-scattered photons. Experimental angular distributions for these two types of events are shown in Fig. 5. The data were approximated by the function Eq. (8). For the initial states, the fitting parameters give $R = 2.435 \pm 0.018$, which is consistent with the Geant4 Monte Carlo simulation, that takes into account the orthogonality of the NaI(Tl) detectors to the setup symmetry axis and range $80^\circ{-}100^\circ$ of photon scattering angles. The modulation factor is equal to $\mu =A^2 = 0.418 \pm 0.003$, which is also in agreement with simulation.

The second plot in Fig. 5 shows the angular distributions for the entire set (classes “a”, “b”, “c” in Fig. 4) of pre-scattered events, excluding backscattered ones. Surprisingly, these events exhibit almost the same azimuthal behavior with a ratio $R = 2.41 \pm 0.10$ and a modulation factor $\mu = 0.414 \pm 0.017$.

**Figure 6:**
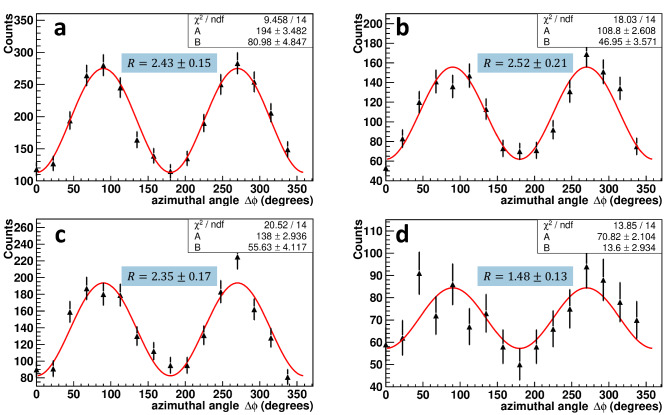
Dependence of coincidence counts in NaI(Tl) detectors on the azimuthal angle between these detectors for four classes ( “a”, “b”, “c” and “d” in Fig. 4) of pre-scattered events. The solid line corresponds to the fitting function Eq. (8). The numbers in the blue area on the graphs indicate the *R* ratio for the corresponding class of events.

To get more information and insight, angular distributions were constructed for all classes of pre-scattered events classified in Fig. 4. The dependencies of the number of scattered photons registered in the NaI(Tl) counters on the azimuthal angle between these photons are shown in Fig. 6.

From the fitting parameters presented in these plots, one can calculate the ratio *R* of the numbers of counts for the perpendicular and parallel orientations of the scattering planes, which are very close to each other, $R_a = 2.43 \pm 0.15$, $R_b = 2.52 \pm 0.21$, $R_c = 2.35 \pm 0.17$ for classes “a”,“b”, “c”, respectively.

The smallest ratio $R_d = 1.48 \pm 0.13$ is observed for class “d” events. We argue that this is due to the partial depolarization of backscattered photons, see “Discussion” section.

### *S*-function in CHSH inequality

The CHSH inequality as a particular variant of Bell’s theorem is used in classical experiments with pairs of entangled optical photons^38–40^. A suitable setup includes two dual-channel polarimeters separating orthogonal linear polarization parallel and perpendicular to some arbitrary $\vec {a}$ and $\vec {b}$ directions for the first and second photons, respectively. By measuring the coincidence rate *R* of photons with different spin orientations, the correlation coefficients can be constructed as:

9 $$\begin{aligned} E(\vec {a},\vec {b})= \frac{R_{a_{\parallel }b_{\parallel }} + R_{a_{\perp }b_{\perp }}-R_{a_{\parallel }b_{\perp }}-R_{a_{\perp }b_{\parallel }}}{R_{a_{\parallel }b_{\parallel }} + R_{a_{\perp }b_{\perp }}+R_{a_{\parallel }b_{\perp }}+R_{a_{\perp }b_{\parallel }}}, \end{aligned}$$

where the indexes $a_{\parallel }$, $a_{\perp }$, $b_{\parallel }$ and $b_{\perp }$ denote the photons with parallel or perpendicular polarization for the direction $\vec {a}$ or $\vec {b}$, respectively.

By measuring the correlation coefficients for four different polarimeter orientations, $\vec {a}, \vec {b}, \vec {a}^\prime , \vec {b}^\prime$, the following function can be composed:

10 $$\begin{aligned} S=E(\vec {a},\vec {b}) - E(\vec {a},\vec {b}^\prime ) + E(\vec {a}^\prime ,\vec {b}) + E(\vec {a}^\prime ,\vec {b}^\prime ). \end{aligned}$$

In the case of maximally entangled photons, *S*-function reaches a maximum of $|S|= 2\sqrt{2}=2.83$ for certain optimal polarimeter orientations:

$$\begin{aligned} (\vec {a},\vec {b}) & =(\vec {a}^\prime ,\vec {b}^\prime )=(\vec {a}^\prime ,\vec {b})=\phi _{opt}\\ (\vec {a},\vec {b}^\prime ) & =(\vec {a},\vec {b})+(\vec {a}^\prime ,\vec {b}^\prime )+(\vec {a}^\prime ,\vec {b}), \end{aligned}$$

where $(\vec {a},\vec {b})$ denotes an angle between vectors $\vec {a}$ and $\vec {b}$, and the optimal azimuthal angles $\phi _{opt}$ are multiples of $\phi =22.5^\circ$. This clearly violates the CHSH inequality $|S|\le 2$ that follows from Bell’s theorem if underlying hidden local variables would exist.

As discussed by Clauser and Shimony^25^, the CHSH inequality is never violated for annihilation photons due to the low efficiency (analyzing power) of Compton polarimeters. To map the real-world experiments with non-ideal polarimeters to the ideal case, the $S$-function is usually normalized to the product of corresponding efficiencies. Moreover, even without such a normalisation, measurements of annihilation photons in initial and pre-scattered states on the same setup allow a direct comparison of the *S*-function for these two types of events.

Since in our setup the angle between adjacent detectors of scattered photons is $22.5^\circ$, the procedure for measuring the correlation coefficients and the *S*-function is straightforward (see also Osuch et al.^26^). The elementary Compton polarimeter consists of a scatterer and two orthogonal detectors of scattered photons, as shown in Fig. 1. To measure the correlation coefficient $E(\vec {a},\vec {b})$, two elementary Compton polarimeters are requested on opposite sides of the setup with orientations $\vec {a}$ and $\vec {b}$. As an example, Fig. 1, left shows a scheme for measuring the correlation coefficient $E(90^{\circ })$. The *S*-function was determined using four polarimeters with orientations $\vec {a}$, $\vec {a}^\prime$ on one side and $\vec {b}$, $\vec {b}^\prime$ on the other. There are 16 Compton polarimeters on each side of the setup. And the count rates for a given correlation coefficient are summed for all relevant combinations of polarimeters.

**Figure 7:**
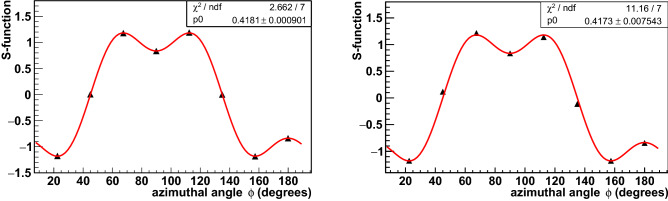
Dependence of the S-function on the relative azimuthal angle between polarimeters for initial (left) and pre-scattered events of class “a” (right). Data points are represented by triangles, error bars are within the symbols. The solid line corresponds to the fitting function Eq. (3).

The measured $S$-functions for initial and pre-scattered annihilation photons are presented in Fig. 7. In the latter case, class “a” of events with an energy deposition below 30 keV in the intermediate GAGG scatterer was selected to ensure the minimum difference from the kinematics of initial photons. The fit of the experimental points by the theoretical function Eq. (3) is shown by the solid line. Surprisingly, the behavior of both $S$-functions is almost identical regardless of the event type. Moreover, the normalizing factors *p*0 are practically identical and coincide with the modulation factors estimated from the angular dependencies of the coincidence counts in the detectors of scattered photons, see Fig. 5. The values of both $S$-functions agree with Monte Carlo simulations based on the Eq. (7) describing the Compton scattering cross section for annihilation photons in the maximally entangled state.

## Discussion

In this paper, we presented a direct comparison of the polarization correlations of annihilation photons in the initial and pre-scattered states obtained in an experiment with unprecedented statistics. Entanglement in the initial state is predicted in a series of theoretical papers^5,8,19,27^, while the state of correlations after interaction/measuring of one of the annihilation photons is under theoretical discussion and has not been experimentally determined before. Meanwhile, for some applications, such as the use of quantum entanglement in the next generation of positron emission tomography (QE-PET), it is necessary to know what happens to the correlations after one of the photons interacts with the environment.

As suggested by Bohm and Hiley^10^, the measurement of one of the two initial photons leads to the collapse of the entangled state (Eq. 1) into a separable mixed state described by Eq. (2), while Bohm and Aharonov^8^ have found that correlations in this state are absent. However, in a recent paper by Hiesmayr and Moskal^27^ it was found that the cross section for Compton scattering of annihilation photons is identical for entangled and separable states Eq. (2). The cross section determines all dependences between the scattered photons, and then any angular distributions must be the same for both quantum states. Later, Caradonna et al.^19^ disagreed with Hiesmayr and Moskal^27^, confirming the results of Bohm and Aharonov^8^ on the absence of angular correlations for separable mixed states.

We used two methods for quantifying correlations. The first method is based on the discovery by Bohm and Aharonov^8^ of the relationship between strong angular correlations of Compton scattered photons and their entanglement. According to authors, a large ratio $R>2$ of the number of photon counts for the perpendicular and parallel orientations of the scattering planes indicates non-local correlations. The second approach involves the CHSH inequality, which is directly related to Bell’s theorem. For annihilation photons, Osuch et al.^26^ measured the *S*-function in this inequality and, after correction for the efficiency of Compton polarimeters, found that it violates the CHSH inequality in accordance with quantum theory.

Our very first measurements of azimuthal angular correlations in the setup without a GAGG scatterer^41^ and further detailed study presented in this work are consistent with the previous experimental results^13–18^ as well as with the theoretical predictions of the entanglement of annihilation photons. Also, our experimental data agree with Monte Carlo simulations based on the Eq. (7) describing the Compton scattering cross section for annihilation photons in the maximally entangled state.

As a new development, we compared Compton scattering of initial annihilation photons and pairs prepared by preliminary scattering of one of the initial photons in the GAGG scintillator before measurements in the polarimeter. This GAGG scintillator, together with the adjacent plastic scatterer, can be regarded as a Compton polarimeter with low analyzing power. For example, 30 keV of energy deposited in GAGG corresponds to a scattering angle of about $20^{\circ }$ with the analyzing power of the respective Compton polarimeter $\sim 7\%$, see Eq. (6). We found that the polarization correlations of annihilation photons in the initial and pre-scattered states are the same for moderately small forward scattering angles and are consistent with the Compton scattering cross section for annihilation photons in the maximally entangled state, see Eq. (7). At the same time, the correlation of the backscattered photon with its pair turns out to be significantly smaller, but even in this case it is still well above the completely depolarized $R=1$ prediction of Bohm and Aharonov^8^.

How can we interpret measured correlations? Recall that at high-energy scattering, the horizontal (vertical) polarization with respect to the scattering plane can change to vertical (horizontal). According to the equation (87.17) in ref.^42^, during backscattering at $E = m_e$ one fifth of photons change horizontal polarization to vertical, and vice versa. Using the cross-sections for the basis set of separable states obtained by Hiesmayr and Moskal^27^, we find that the modulation factor reduces by a factor of 3/5 . We should expect $R \approx 1.66$ for such a depolarized state, which is consistent with the experimental result, Fig. 6d. On the other hand, at scattering angles below $40^\circ$ the probability of $V\leftrightarrow H$ transitions are below 1%. In other words, the polarisation state of photons changes insignificantly. This may explain why we do not observe a difference between the correlations of initial and forward pre-scattered events within experimental accuracy.

We hope that our experimental results will help clarify the current theoretical controversy. In any case, the results obtained are directly related to the applicability of quantum entanglement in next-generation positron emission tomography (QE-PET). The pre-scattered events in our experiment accurately reproduce the scattering background in the body, which limits the image quality^28^. The similarity of correlations for the initial and pre-scattered events indicates that most of the scattered backgrounds cannot be rejected by constraints on the angular distribution of scattered photon pairs^43^. At the same time, the random background obviously has no angular correlations and can be suppressed by applying appropriate kinematic cuts.

## Data Availability

The analysed experimental data presented in the manuscript would be made available upon request.
